# Supplementary figures and images for: Circular RNA expression and regulatory network prediction in posterior cingulate astrocytes in elderly subjects
Source: BMC Genomics. 2018 May 9;19:340. doi: 10.1186/s12864-018-4670-5 (PMC5941680; doi:10.1186/s12864-018-4670-5)

Additional file 3: Figure S1

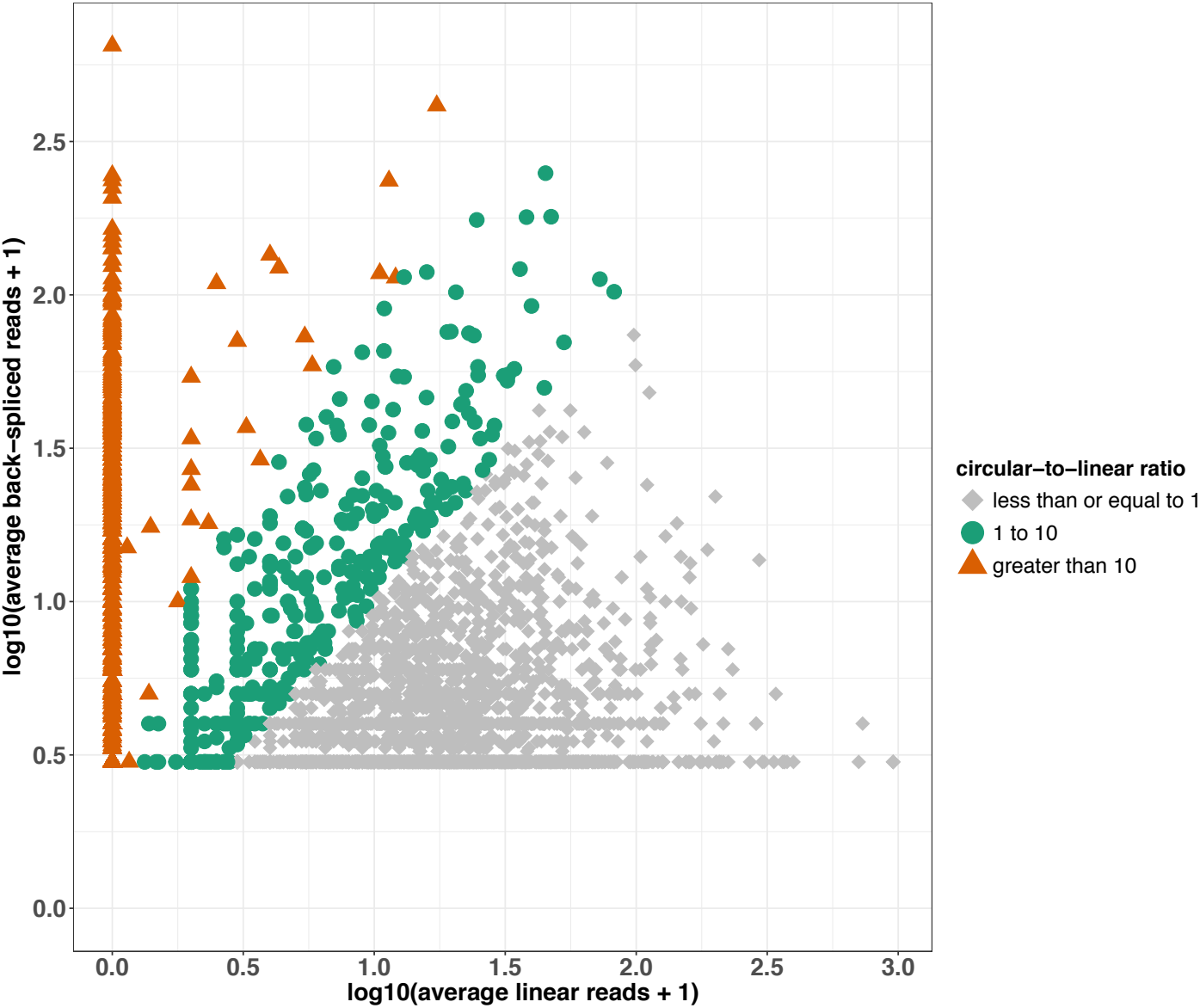

Supplement: Supplementary file 3 — Figure S1. Circular-to-linear ratios. Ratio of average back-spliced reads to average linearly spliced reads for all detected circRNAs. (PDF 1075 kb) [file 12864_2018_4670_MOESM3_ESM.pdf]

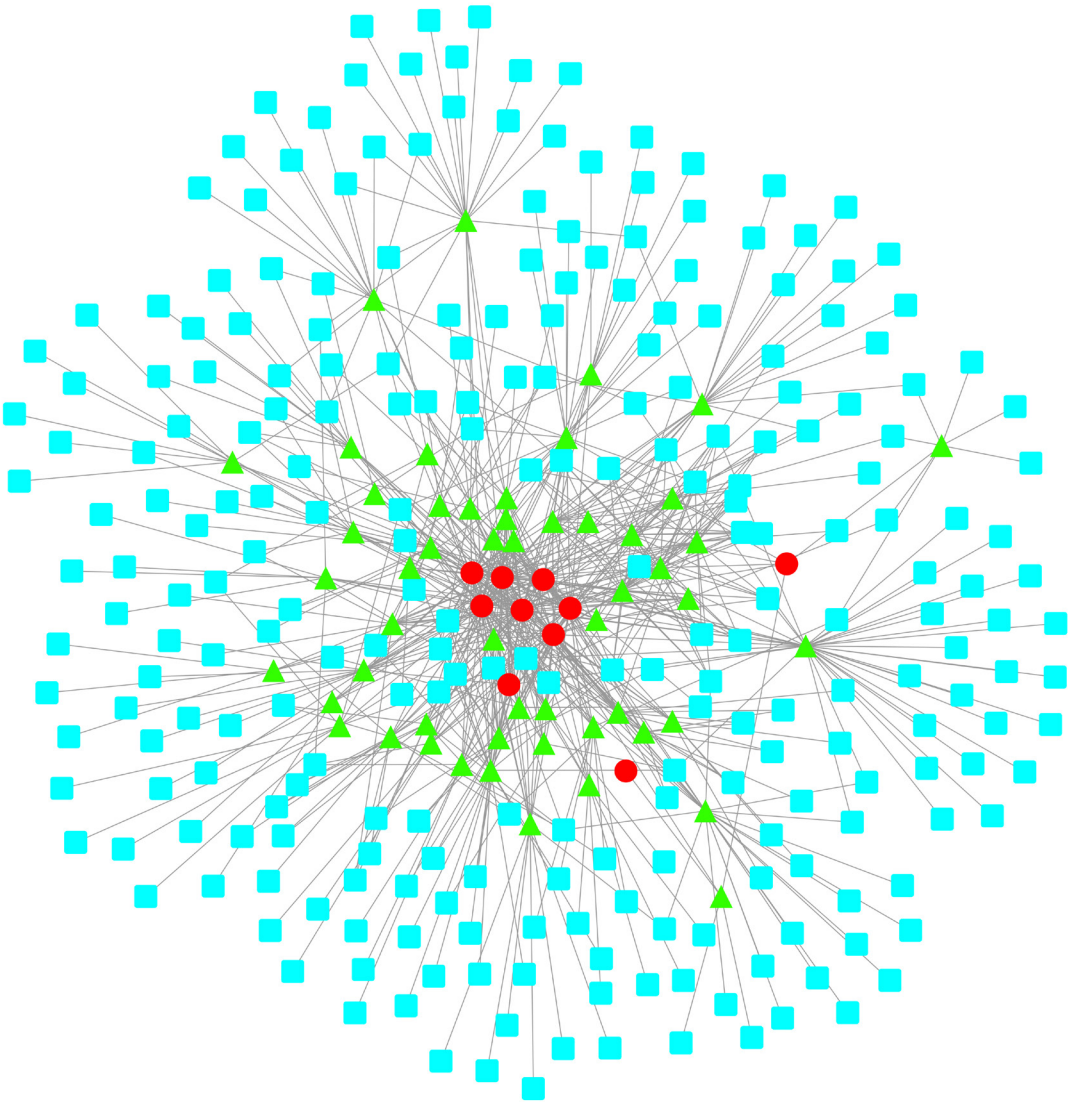

● circRNA    ▲ miRNA    ■ gene

Supplement: Supplementary file 6 — Figure S2. Low stringency circRNA-miRNA-mRNA regulatory network. Network of circRNA-miRNA-mRNA regulation for those circRNA-miRNA interactions predicted by both RNAHybrid and miRanda, with miRanda match scores > = 150 and mRNA targets with differential expression (uncorrected P < 0.05). Red circular nodes: circRNAs, green triangular nodes: miRNAs, blue square nodes: genes. (PDF 771 kb) [file 12864_2018_4670_MOESM6_ESM.pdf]

Additional file 7: Figure S3

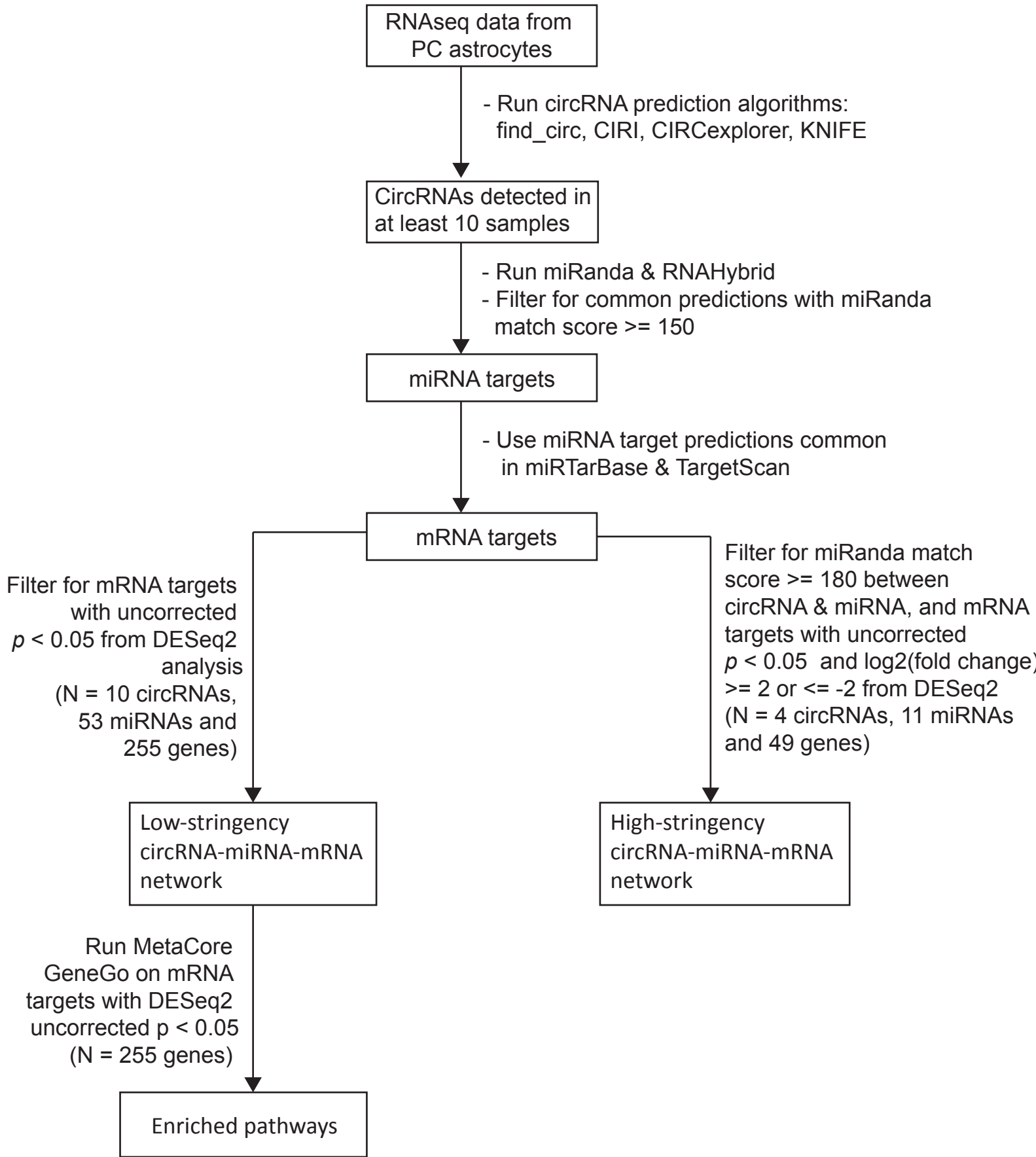

Supplement: Supplementary file 7 — Figure S3. Computational workflow outline and filtering criterion. PC, posterior cingulate; RNAseq, RNA sequencing; circRNA, circular RNA; miRNA, microRNA; mRNA, messenger RNA. (PDF 928 kb) [file 12864_2018_4670_MOESM7_ESM.pdf]
